# Supplementary figures and images for: Maternal obesity induces activator protein 1‐mediated inflammatory response to impair embryonic neurogenesis
Source: J Physiol. 2026 Mar 13;604(7):3159–74. doi: 10.1113/JP289326 (PMC13039269; doi:10.1113/JP289326)

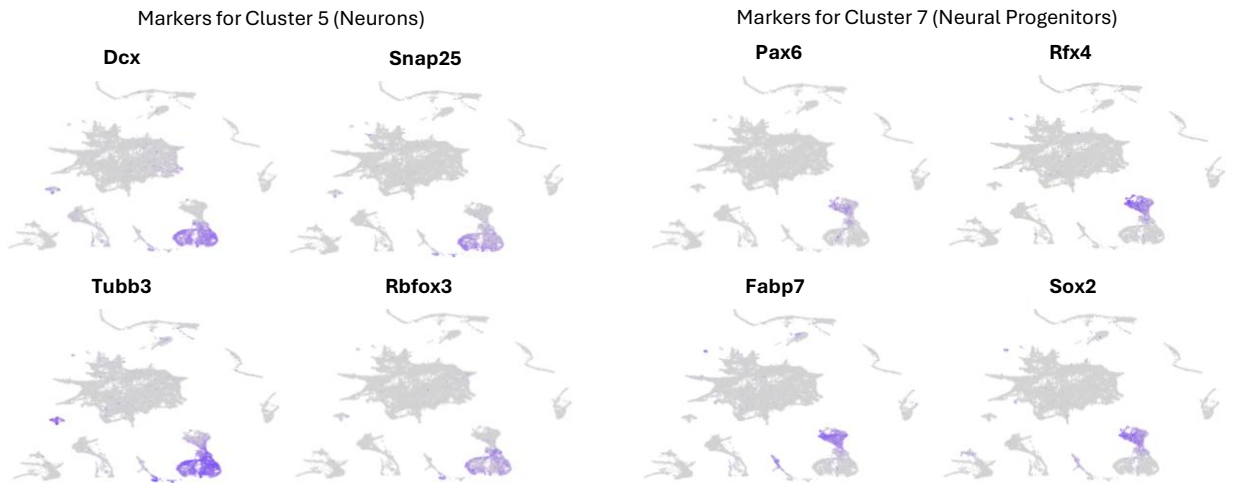

Figure A1. Markers used for identification neurons and neural progenitors.

Supplement: Supplementary file 2 — Figure A1. Markers used for identification neurons and neural progenitors. [file TJP-604-3159-s001.pdf]
