# Supplementary material for: Maternal obesity induces activator protein 1‐mediated inflammatory response to impair embryonic neurogenesis
Source: J Physiol. 2026 Mar 13;604(7):3159–74. doi: 10.1113/JP289326 (PMC13039269; doi:10.1113/JP289326)
Supplement: Supplementary file 3 — Figure A2. Maternal serum levels of LDL (low‐density lipoprotein), HDL (high‐density lipoprotein), TG (triglyceride), glucose, insulin and TNF‐α (tumour necrosis factor‐α). Obese dams exhibited significantly elevated lipid levels (LDL, HDL, TG) and increased circulating TNF‐α compared to controls (CONs), whereas glucose and insulin levels were not significantly different between groups. Data are presented as mean ± SD, and each dot represents one dam (n = 6). P‐value in CON versus MO (maternal obesity) using unpaired Student's t test. [file TJP-604-3159-s003.pdf]

Fig. A2

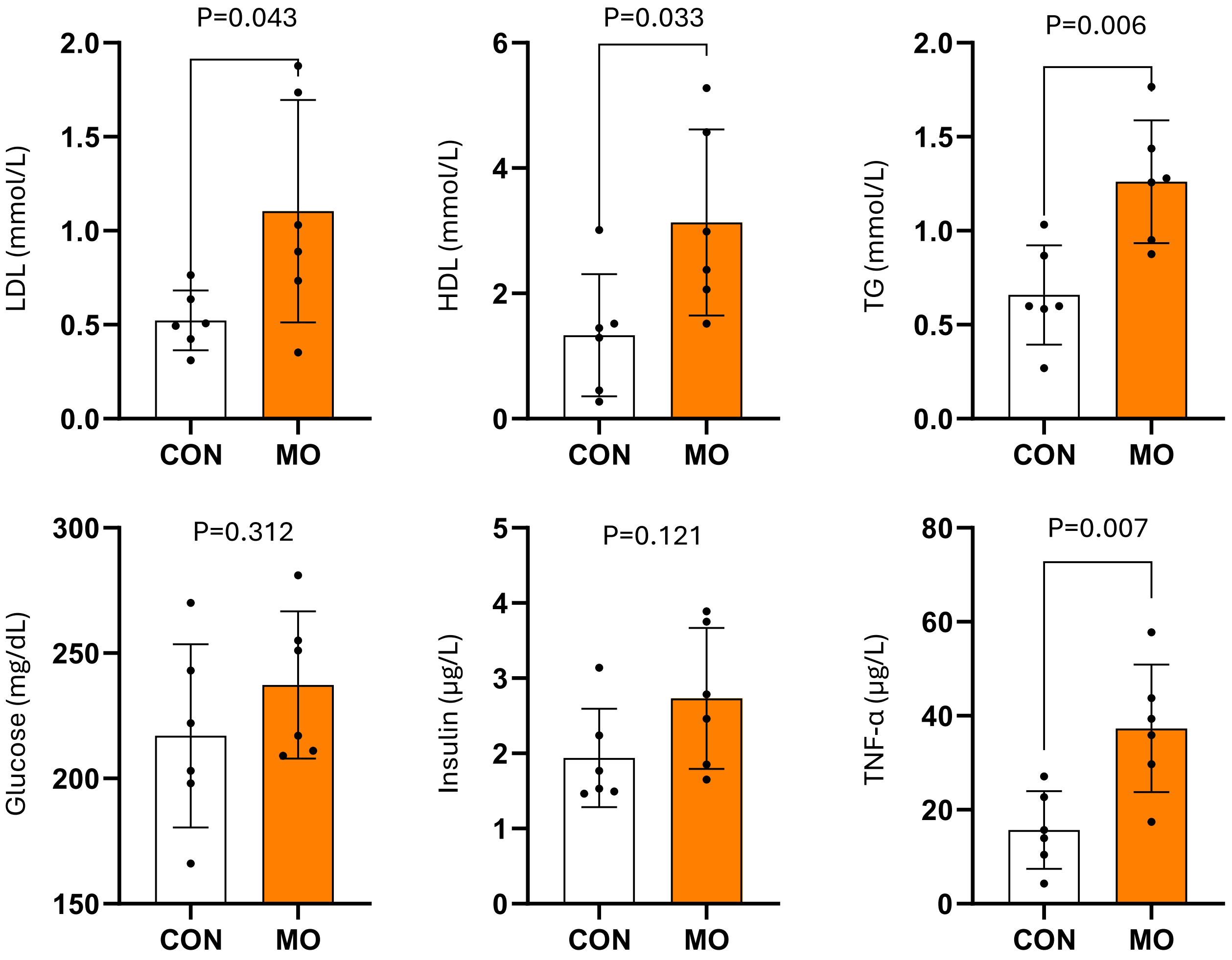

Figure A2. Maternal serum levels of LDL (low-density lipoprotein), HDL (high-density lipoprotein), TG (triglyceride), glucose, insulin and TNF-α (tumour necrosis factor-α). Obese dams exhibited significantly elevated lipid levels (LDL, HDL, TG) and increased circulating TNF-α compared to controls (CONs), whereas glucose and insulin levels were not significantly different between groups. Data are presented as mean ± SD, and each dot represents one dam ($n = 6$). P-value in CON vs. MO (maternal obesity) using unpaired Student's t test.
